# Completely Resected N0 Non-Small Cell Lung Cancer: Prognostic Factors Affecting Long-Term Survival

**DOI:** 10.1155/2013/175304

**Published:** 2013-08-29

**Authors:** Apichat Tantraworasin, Somcharoen Saeteng, Nirush Lertprasertsuke, Nuttapon Arayawudhikule, Choosak Kasemsarn, Jayanton Patumanond

**Affiliations:** ^1^General Thoracic Unit, Department of Surgery, Faculty of Medicine, Chiang Mai University Hospital, Chiang Mai 50200, Thailand; ^2^Department of Pathology, Faculty of Medicine, Chiang Mai University Hospital, Chiang Mai 50200, Thailand; ^3^Cardiovascular Thoracic Unit, Department of Surgery, Lampang Hospital, Lampang 52000, Thailand; ^4^Cardiovascular Thoracic Unit, Department of Surgery, Chest Institute, Nonthaburi 11000, Thailand; ^5^Department of Community Medicine, Faculty of Medicine, Chiang Mai University Hospital, Chiang Mai 50200, Thailand

## Abstract

*Background.* Although early stage non-small cell lung cancer (NSCLC) has an excellent outcome and correlated with good long-term survival, up to 15 percent of patients still relapse postoperatively and die. This study is conducted to identify prognostic factors that may affect the long-term survival in completely resected N0 NSCLC. *Methods.* Medical records of 124 patients with completely resected N0 NSCLC were retrospectively reviewed. Prognostic factors affecting long-term survival were analyzed by the Kaplan-Meier method and Cox proportional hazards analysis. *Results.* Overall five-year survival rate was 48 percent. Multivariable analysis revealed stage of disease, tumor necrosis, tumor recurrence, brain metastasis, adrenal metastases, and skin metastases as significant prognostic factors affecting long-term survival. The hazard ratio (HR) of tumor necrosis, tumor recurrence, brain metastasis, adrenal metastases, and skin metastases was 2.0, 2.3, 7.6, 4.1, and 8.3, respectively, and all *P* values were less than 0.001. *Conclusions*. Our study shows stage of disease, tumor necrosis, tumor recurrence, brain metastasis, adrenal metastasis, and skin metastasis as the independent prognostic factors of long-term survival in pathological N0 NSCLC. Early stage NSCLC patients without nodal involvement or presented with tumor necrosis should benefit from adjuvant chemotherapy, and sites of metastasis could predict the long-term survival as described.

## 1. Introduction

The curative treatment for early stage non-small cell lung cancer (NSCLC) patients without nodal involvement is surgery alone; however, some pathological characteristics may be associated with poor prognostic of long-term survival. Overall 5-year survival rates in completely resected stages IA and IB NSCLC range from 67 to 89% and from 57 to 75%, respectively; therefore, there are some poor prognostic factors that affect the overall survival despite presenting in the same stage [1–4]. Recently, some prognostic factors such as intratumoral blood vessel invasion (IVI), intratumoral lymphatic invasion (ILI), visceral pleural invasion, tumor size, and serum level of carcinoembryonic antigen (CEA), have been proposed in order to identify poor prognostic factors providing beneficial adjuvant chemotherapy for stage I NSCLC patients. Some studies showed that the strong poor prognostic factors in these patients are IVI and ILI [1, 5–7]. The purpose of this study is to identify prognostic factors associated with poor prognosis in overall survival in completely resected N0 NSCLC patients.

## 2. Patients and Methods

Between January 2008 and December 2011, 232 patients underwent anatomical resection (lobectomy, sleeve lobectomy, bilobectomy, and pneumonectomy) with systematic mediastinal lymph node dissection at Chiang Mai University Hospital, Chiang Mai, Thailand. One-hundred twenty-four patients were enrolled in this study because of no pathological lymph node involvement. We retrospectively reviewed these 124 cases from the medical recording system with regard to patient characteristics, signs and symptoms, tumor pathologic report, and follow-up status to examine the prognostic factors affecting the overall survival. In the preoperative evaluation, standard laboratory tests such as complete blood count (CBC), liver and renal function test, electrolyte, chest film, computed tomographic (CT) scan of the thorax including the upper abdomen, pulmonary function test, and bronchoscopy were performed on all of the patients. Bone scan and CT of the brain were obtained only in patients who had evidence of bone or brain metastasis. Patients with evidence of residual tumor at the resection margin (five patients) or who died within the first 28 days of the surgery (postoperative mortality) (three patients) or had single brain metastasis who underwent a craniectomy to remove tumor before pulmonary resection without nodal involvement (five patients) were all excluded. All of these patients did not received adjuvant chemotherapy.

The surgical procedures consisted of 114 lobectomies (91.9%) and 10 bilobectomies (8.1%). Systematic mediastinal lymph node dissection was performed in all cases, and all nodal stations were labeled according to the staging manual in thoracic oncology [8].

All excised specimens were formalin-fixed and sliced at ten-millimeter intervals. Histopathologic examination was performed by the same pathologist. Pathological staging was determined according to the IASLC TNM staging classification of NSCLC [9]. Histological subtypes of lung cancer were determined according to World Health Organization classification [10]. Visceral pleural invasion was defined as tumor extending beyond the elastic layer of the visceral pleura and/or exposed on the pleural surface but did not involve the parietal pleura. IVI and ILI were defined as tumor cells identifiable in the blood vessel lumen and lymphatic lumen, respectively. Tumor necrosis was defined as coagulative necrosis identifiable in the tumor. Tumor involvement of epineurium was defined as perineural invasion [11].

All patients were actively followed postoperatively for two weeks and for three- to six-month intervals for the first two years, and then yearly thereafter with a CT scan of the chest and upper abdomen. If patients developed signs or symptoms that correlated with tumor recurrence or metastasis, they would be worked up according to their signs or symptoms (i.e., CT brain or bone scan). Tumor recurrence was defined as evidence of tumor within the same lobe, the hilum, or the mediastinal lymph nodes (locoregional recurrence) or evidence of tumor in another lobe or elsewhere outside the hemithorax (distant recurrence). The interval to recurrence was defined as the interval between the time of the operation and the discovery of the recurrence by means of either imaging or cytopathological examination. Patients who developed brain metastasis were treated with whole brain radiation (multiple brain metastasis) or tumor excision (single brain metastasis and no other site of metastasis). Standard chemotherapy and radiotherapy were used when having an indication.

The overall survival curves were estimated using the Kaplan-Meier method. Prognostic factors for overall survival were investigated univariately and multivariately by using a Cox multivariable regression model stratified by age. Univariable prognostic factors significant at the 0.05 level were considered for the multivariable models. Values of *P* < 0.05 were considered significant. All tests were two-tailed and performed with commercial statistical software STATA version 11.0 (STATA Corp., CS, USA).

This study was reviewed and approved by the Research Ethics Committee, Faculty of Medicine, Chiang Mai University, Chiang Mai, Thailand.

## 3. Results

Patient's characteristic and pathological reports of patients are shown in Tables 1 and 2. The study cohort included 53 women and 71 men with a mean age of 61.8 years (range 24–83 years). Most common clinical presentation is chronic cough and hemoptysis. Forty-one percent of these patients are asymptomatic. Lobectomy is the most common procedure (91.9%). Histopathology was adenocarcinoma in 70 patients (56.5%), squamous cell carcinoma in 33 patients (26.6%), and others in 21 patients (16.9%). Pathological stages IA, IB, IIA, and IIB were 33 (26.6%), 45 (36.3%), 21 (16.9%), and 25 (20.2%), respectively. A total of 48 patients (38.7%) had a tumor necrosis, 23 patients (18.6%) had a visceral pleural invasion, 91 patients (73.4%) had an ILI, and 40 patients (32.3%) had an IVI. Mean postoperative follow-up time was 29.1 months (range 1.6–144.9 months). There were 47 deaths (37.9%) during the follow-up period.

Univariable analysis demonstrated that there are eight significant prognostic factors which affect the overall survival (*P* < 0.05): age ≥ 70 years, staging of lung cancer, tumor necrosis, tumor recurrence, brain metastasis, adrenal metastasis, and skin metastasis (Table 3). No significant difference was seen for gender, smoking, histologic grading, histologic cell type, visceral pleural invasion, intratumoral blood vessel invasion, intratumoral lymphatic invasion, and other sites of metastasis.

Multivariable analysis stratified by age demonstrated stage of disease (or tumor size, because of N0 and M0, stage of disease represented the tumor size), tumor necrosis, tumor recurrence, brain metastasis, adrenal metastasis, and skin metastasis as strong independent prognostic factors of overall survival. The hazard ratio of death was 4.6 (95% confidence interval (CI), 2.1–10.3; *P* < 0.001 for stage IIA), 4.0 (95% CI, 3.1–5.1; *P* < 0.001  for stage IIB (both stages comparing with stage IA)), 2.0 (95% CI, 1.5–2.8; *P* < 0.001 for tumor necrosis), 2.3 (95% CI, 1.6–3.3; *P* < 0.001 for tumor recurrence), 7.6 (95% CI, 4.0–14.2; *P* < 0.001 for brain metastasis), 4.1 (95% CI, 3.0–5.7; *P* < 0.001 for adrenal metastasis), and 8.3 (95% CI, 2.6–26.4; *P* < 0.001 for skin metastasis) as shown in Table 4.

**Figure 1:**
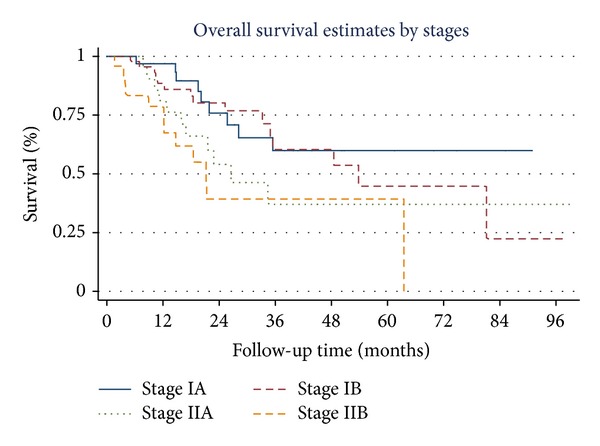
Survival curves by stages.

**Figure 2:**
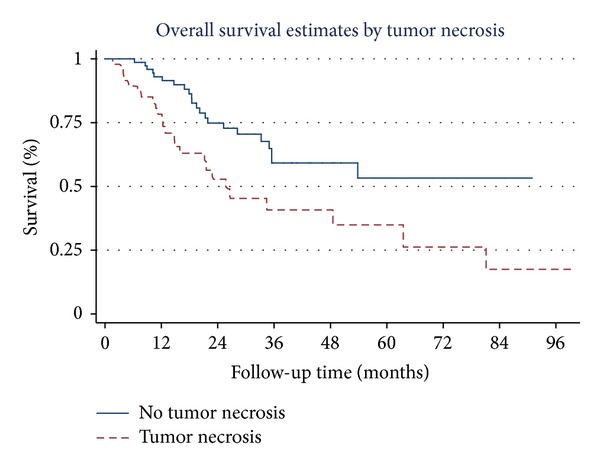
Survival curves by tumor necrosis.

**Figure 3:**
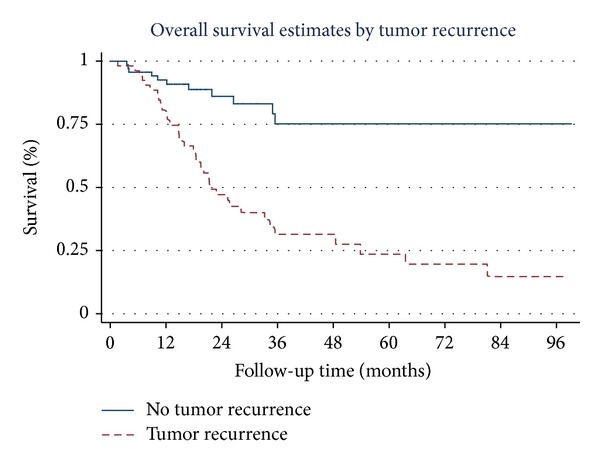
Survival curves by tumor recurrence.

**Figure 4:**
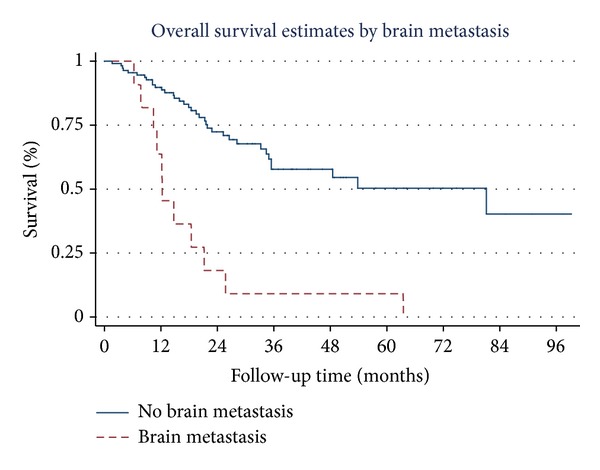
Survival curves by brain metastases.

**Figure 5:**
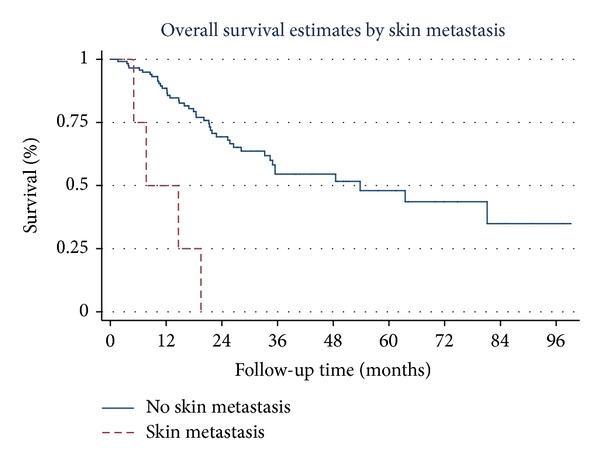
Survival curves by skin metastases.

**Figure 6:**
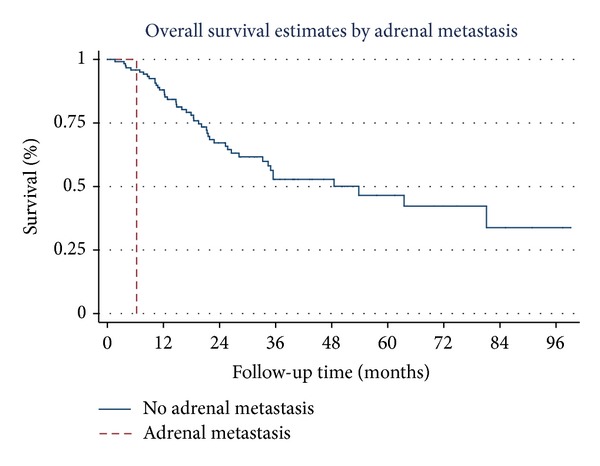
Survival curves by adrenal metastases (One patient developed adrenal metastasis during follow-up CT-scan 3 month after surgery and then death in 3 months later).

Overall survival estimates curves by stage of NSCLC, tumor necrosis, tumor recurrence, brain metastasis, skin metastasis, and adrenal metastasis are shown in Figures 1, 2, 3, 4, 5, and 6. Overall 2-year and 5-year survival rates in patients with and without prognostic factors were shown in Table 5. Patients who had any prognostic factor had significant shorter overall 2-year and 5-year survival than patients who did not have.

There was only one patient that had adrenal metastasis, and this patient died within 2 months after diagnosed adrenal metastasis because one month after that he had lung metastasis and developed respiratory failure.

## 4. Discussion 

The aim of this study was to identify independent prognostic factors of long-term survival in patients diagnosed early stage NSCLC (no nodal involvement) who underwent completely anatomical resection and mediastinal lymph node dissection. The principle findings were stage of disease or tumor size, tumor necrosis, tumor recurrence, brain metastasis, skin metastasis, and adrenal metastasis that are prognostic factors of poor long-term survival.

Recently, several prognostic factors for early stage (stage I and stage II without nodal involvement) NSCLC have been identified including intratumoral blood vessel invasion (IVI) [12–15], intratumoral lymphatic invasion (ILI) [1, 11], mitotic index and nuclear atypia [15, 16], visceral pleural invasion [17], and degree of histologic differentiation [16, 18, 19]. Regarding intratumoral blood vessel and lymphatic invasion, many previous studies showed that these factors have been considered as prognostic factors [12, 20, 21]. Yilmaz et al. [11] reported that lymphovascular invasion can show a higher risk of mortality in completely resected NSCLC. Pechet et al. [12] summarized that presentation of arterial invasion in stage I NSCLC patients was adversely associated with poor survival (hazard ratio (HR) of 3.5 and *P* value < 0.001). Miyoshi et al. [21] and Shoji et al. [13] concluded that IVI was independent prognostic factor in pathological stage I NSCLC patients. In contrast, some studies did not show the relevant prognostic factors [22]. In our study, IVI and ILI have not been shown as prognostic factors. This study also did not demonstrate visceral pleural invasion as poor prognostic factor of overall survival like other previous studies [11, 23].

Maximum tumor diameter is a valuable prognostic factor based on gross specimen [19]. In our study, stage of disease (only T is affected because no nodal involvement) is one of the poor prognostic factors. The overall survival of patients who diagnosed with stage II was significantly shorter than that of diagnosed with stage I. This result was the same as the study of Harada et al. [20] and other previous studies [24].

There were no previous studies demonstrating that the tumor necrosis was the poor prognostic factor. In our study, presenting tumor necrosis in stages IA, IB, IIA, and IIB was 12.1%, 33.3%, 66.7%, and 60.0%, respectively. As we have noticed, large tumors have more percentage of tumor necrosis than the small ones. One of the possible reasons why big tumors had more tumor necrosis was due to less vascular supply or blood vessels in the central part of the tumor; therefore, large tumors had more chances for presenting with tumor necrosis than the small ones. In this study, the multivariable Cox regression analysis demonstrated that tumor necrosis is one of the poor prognostic factors of overall survival.

We already knew that tumor recurrence was poor prognostic factor of overall survival. Our results confirm that theory, however we found that brain metastasis, adrenal metastasis and skin metastasis were poor prognostic factors of overall survival comparing with other sites of tumor recurrence. There were no previous studies that show correlation between site of tumor recurrence and overall survival in completely resected early stage NSCLC patients.

Limitation of this study was retrospective nature and small sample size. Some prognostic factors that did not show poor prognostic factors may be because of small sample size. We also believe that large-scale studies are necessary to clarify the result of this study.

## 5. Conclusion

This study demonstrated that T stage of disease, tumor necrosis, tumor recurrence, brain metastasis, adrenal metastasis, and skin metastasis are poor prognostic factors of overall survival in completely resected early stage NSCLC patients. Patients who diagnosed more than pathologic stage IA or presented with tumor necrosis may gain survival benefits from adjuvant chemotherapy.

## Figures and Tables

**Table 1:** Patient's characteristics in completely resected NSCLC without nodal involvement.

Characteristics	*n* (%)
Age (year)	
<60	45 (36.3)
60–69	44 (35.5)
≥70	35 (28.2)
Mean ± SD (range)	61.8 ± 11.0 (24–83)
Gender	
Female	53 (42.7)
Male	71 (57.3)
Smoking	
Never smoked	38 (30.7)
Stopped smoking	80 (64.5)
Still smoking	6 (4.8)
Mean packed year ± SD	20.0 ± 18.1
Family history of malignancy	6 (4.8)
Underlying diseases	
Chronic lung disease	17 (13.7)
Diabetic mellitus	13 (10.5)
Essential hypertension	46 (37.1)
Dyslipidemia	21 (16.9)
Symptoms	
Hemoptysis	48 (38.7)
Chronic cough	50 (40.3)
Poor appetite	16 (12.9)
Significant weight loss	29 (23.4)
Chest pain	11 (8.9)
Dyspnea	24 (19.4)
Asymptomatic	51 (41.1)

**Table 2:** Treatments and pathological reports.

Parameters	*n* (%)
Surgical procedures	
Lobectomy	114 (91.9)
Bilobectomy (RUL and RML)	1 (0.8)
Bilobectomy (RLL and RML)	9 (7.3)
Histologic types	
Adenocarcinoma	70 (56.5)
Squamous cell carcinoma	33 (26.6)
Bronchioloalveolar carcinoma	10 (8.1)
Large cell carcinoma	5 (4.0)
Neuroendocrine tumor	3 (2.4)
Adenoid cystic CA	2 (1.6)
Adenosquamous	1 (0.8)
Tumor grading	
Well differentiated	41 (33.1)
Moderately differentiated	45 (36.3)
Poorly differentiated	24 (19.4)
Undifferentiated	5 (4.0)
Mucinous type (BAC)	5 (4.0)
Nonmucinous type (BAC)	4 (3.2)
Pathological staging	
IA	33 (26.6)
IB	45 (36.3)
IIA	21 (16.9)
IIB	25 (20.2)
Tumor diameter (cm)	4.8 ± 2.7
≤3	33 (26.6)
>3	91 (73.4)
Tumor necrosis	48 (38.7)
Visceral pleural invasion	23 (18.6)
Neural invasion	2 (1.6)
Intratumoral lymphatic invasion	91 (73.4)
Intratumoral blood vessel invasion	40 (32.3)
Follow-up time (months)	29.1 ± 24.6
Tumor recurrence	53 (42.7)
Death	47 (37.9)

**Table 3:** Univariable analysis of overall survival in completely resected NSCLC without nodal involvement by Cox proportional hazard model.

Parameters	Hazard ratio	95% confident interval	*P* value
Age (year)			
<60	Reference		
60–69	1.7	0.8–3.7	0.157
≥70	2.5	1.2–5.3	0.018
Male	1.4	0.8–2.5	0.258
Smoking	1.3	0.8–2.2	0.257
COPD	1.8	0.9–3.6	0.099
Histologic grading	1.0	0.9–1.2	0.764
Histologic cell type	1.0	0.9–1.3	0.466
Staging of lung cancer			
IA	Reference		
IB	1.4	0.6–3.1	0.376
IIA	2.4	0.9–5.7	0.057
IIB	2.9	1.2–7.0	0.015
Tumor size >3 cm	1.9	0.9–4.0	0.078
Visceral pleural invasion	1.2	0.6–2.4	0.685
Intratumoral vascular invasion	1.5	0.8–2.7	0.195
Intratumoral lymphatic invasion	1.5	0.7–3.1	0.276
Tumor necrosis	2.2	1.2–3.9	0.007
Tumor recurrence	4.7	2.4–9.3	<0.001
Lung metastasis	1.3	0.7–2.5	0.351
Pleural metastasis	4.2	0.9–17.6	0.053
Bone metastasis	1.7	0.6–4.7	0.323
Brain metastasis	5.2	2.6–10.3	<0.001
Liver metastasis	1.1	0.1–7.7	0.958
Chest wall metastasis	4.9	0.7–36.8	0.119
Adrenal metastasis	24.1	2.8–205.9	0.004
Renal metastasis	8.7	1.1–66.5	0.037
Skin metastasis	7.9	2.7–22.9	<0.001

**Table 4:** Significant determinants of overall survival in completely resected NSCLC without nodal involvement by Cox proportional hazard model*.

Parameters	Hazard ratio	95% confident interval	*P* value
Staging of lung cancer			
IA	Reference		
IB	1.6	0.9–2.8	0.135
IIA	4.6	2.1–10.3	<0.001
IIB	4.0	3.1–5.1	<0.001
Tumor necrosis	2.0	1.5–2.8	<0.001
Tumor recurrence	2.3	1.6–3.3	<0.001
Brain metastasis	7.6	4.0–14.2	<0.001
Adrenal metastasis	4.1	3.0–5.7	<0.001
Skin metastasis	8.3	2.6–26.4	<0.001

*Stratified by age.

**Table 5:** The five-year survival of patients with and without poor prognostic factors.

Prognostic factors	2-year survival (%)	5-year survival (%)
Stage of lung cancer		
Stage IA	76.8	61.7
Stage IB	76.8	44.7
Stage IIA	54.0	37.1
Stage IIB	43.0	43.0
Tumor necrosis		
No	75.3	54.6
Yes	54.1	37.3
Tumor recurrence		
No	86.6	76.5
Yes	47.1	23.6
Brain metastasis		
No	73.0	52.3
Yes	18.2	9.1
Skin metastasis		
No	70.0	50.0
Yes	0.0	0.0
Adrenal metastasis		
No	67.9	48.3
Yes	0.0	0.0

## References

[1] Liu W, Morito D, Takashima S (2011). Identification of RNF213 as a susceptibility gene for moyamoya disease and its possible role in vascular development. *PLoS ONE*.

[2] Goya T, Asamura H, Yoshimura H (2005). Prognosis of 6644 resected non-small cell lung cancers in Japan: a Japanese lung cancer registry study. *Lung Cancer*.

[3] Inoue K, Sato M, Fujimura S (1998). Prognostic assessment of 1310 patients with non-small-cell lung cancer who underwent complete resection from 1980 to 1993. *Journal of Thoracic and Cardiovascular Surgery*.

[4] Asamura H, Goya T, Koshiishi Y (2008). A Japanese lung cancer registry study: prognosis of 13,010 resected lung cancers. *Journal of Thoracic Oncology*.

[5] Matsuguma H, Nakahara R, Igarashi S (2008). Pathologic stage I non-small cell lung cancer with high levels of preoperative serum carcinoembryonic antigen: clinicopathologic characteristics and prognosis. *Journal of Thoracic and Cardiovascular Surgery*.

[6] Tsuchiya T, Akamine S, Muraoka M (2007). Stage IA non-small cell lung cancer: vessel invasion is a poor prognostic factor and a new target of adjuvant chemotherapy. *Lung Cancer*.

[7] Ogawa J-I, Tsurumi T, Yamada S, Koide S, Shohtsu A (1994). Blood vessel invasion and expression of sialyl Lewis(x) and proliferating cell nuclear antigen in Stage I non-small cell lung cancer: relation to postoperative recurrence. *Cancer*.

[8] Goldstraw P (2009). *Staging Manual in Thoracic Oncology*.

[9] Goldstraw P (2009). Editorial: The 7th edition of TNM in lung cancer: what now?. *Journal of Thoracic Oncology*.

[10] Travis WD, Brambilla E, Muller-Hermelink HK, Harris CC (2004). *World Health Organization Classification of Tumors. Pathology and Genetics of Tumours of the Lung, Pleura, Thymus and Heart*.

[11] Yilmaz A, Duyar SS, Cakir E (2011). Clinical impact of visceral pleural, lymphovascular and perineural invasion in completely resected non-small cell lung cancer. *European Journal of Cardio-Thoracic Surgery*.

[12] Pechet TTV, Carr SR, Collins JE, Cohn HE, Farber JL (2004). Arterial invasion predicts early mortality in stage I non-small cell lung cancer. *Annals of Thoracic Surgery*.

[13] Shoji F, Haro A, Yoshida T (2010). Prognostic significance of intratumoral blood vessel invasion in pathologic stage IA non-small cell lung cancer. *Annals of Thoracic Surgery*.

[14] Schuchert MJ, Schumacher L, Kilic A (2011). Impact of angiolymphatic and pleural invasion on surgical outcomes for Stage i non-small cell lung cancer. *Annals of Thoracic Surgery*.

[15] Takise A, Kodama T, Shimosato Y, Watanabe S, Suemasu K (1988). Histopathologic prognostic factors in adenocarcinomas of the peripheral lung less than 2 cm in diameter. *Cancer*.

[16] Kurokawa T, Matsuno Y, Noguchi M, Mizuno S, Shimosato Y (1994). Surgically curable “early” adenocarcinoma in the periphery of the lung. *American Journal of Surgical Pathology*.

[17] Maeda R, Yoshida J, Ishii G, Hishida T, Nishimura M, Nagai K (2011). Risk factors for tumor recurrence in patients with early-stage (stage I and II) non-small cell lung cancer: patient selection criteria for adjuvant chemotherapy according to the seventh edition TNM classification. *Chest*.

[18] Shimada Y, Saji H, Yoshida K (2012). Pathological vascular invasion and tumor differentiation predict cancer recurrence in stage IA non-small-cell lung cancer after complete surgical resection. *Journal of Thoracic Oncology*.

[19] Kobayashi N, Toyooka S, Soh J (2007). Risk factors for recurrence and unfavorable prognosis in patients with stage I non-small cell lung cancer and a tumor diameter of 20 mm or less. *Journal of Thoracic Oncology*.

[20] Harada M, Hato T, Horio H (2011). Intratumoral lymphatic vessel involvement is an invasive indicator of completely resected pathologic stage i non-small cell lung cancer. *Journal of Thoracic Oncology*.

[21] Miyoshi K, Moriyama S, Kunitomo T, Nawa S (2009). Prognostic impact of intratumoral vessel invasion in completely resected pathologic stage I non-small cell lung cancer. *Journal of Thoracic and Cardiovascular Surgery*.

[22] Sayar A, Turna A, Solak O, Kiliçgün A, Ürer N, Gürses A (2004). Nonanatomic prognostic factors in resected nonsmall cell lung carcinoma: the importance of perineural invasion as a new prognostic marker. *Annals of Thoracic Surgery*.

[23] Taylor MD, Nagji AS, Bhamidipati CM (2012). Tumor recurrence after complete resection for non-small cell lung cancer. *Annals of Thoracic Surgery*.

[24] Kim ES, Lee IJ, Bae Y-A, Lee J-W, Im HJ, Jeon YH (2009). Evaluation of factors relating to tumor recurrence and survival after resection of lung cancer. *Acta Radiologica*.

